# Psychotropic drug repurposing for COVID-19: a systematic review

**DOI:** 10.1192/j.eurpsy.2022.857

**Published:** 2022-09-01

**Authors:** U. Isayeva, G. Fico, S. Gomes-Da-Costa, M. Sagué Villavella, A. Gimenez, M. Manchia, A. Murru

**Affiliations:** 1 University of Cagliari, Section Of Psychiatry, Department Of Medical Sciences And Public Health, Cagliari, Italy; 2 University of Cagliari, Division Of Neuroscience, Department Of Biomedical Sciences, Cagliari, Italy; 3 University of Barcelona, Bipolar And Depressive Disorders Unit, Institute Of Neuroscience, Hospital Clinic, Barcelona, Spain; 4 Dalhousie University, Department Of Pharmacology, Halifax, Canada

**Keywords:** Antidepressants, Antipsychotics, drug repurposing, Covid-19

## Abstract

**Introduction:**

Recently, several antidepressants, mood stabilizers, and antipsychotics have been suggested to have favorable effects in the treatment of COVID-19.

**Objectives:**

The aim of this systematic review was to collect evidence from preclinical and clinical studies concerning the scientific evidence for the repurposing of psychotropic drugs in COVID-19 treatment.

**Methods:**

Two independent authors searched PubMed-MEDLINE, Scopus, PsycInfo, Clinical Trial Registration Site US (ClinicalTrials.gov) databases, and reviewed the reference lists of articles for eligible articles published up to May 31st, 2021. All preclinical and clinical studies on the effect of any psychotropic drug on Sars-CoV-2 or patients with COVID-19 were included. The Newcastle-Ottawa scale was used for the quality assessment of clinical studies. This systematic review adheres to the PRISMA guidelines.

**Results:**

22 studies were included in the synthesis: 9 clinical studies, 9 preclinical studies, and 4 computational studies. The use of antidepressants, both SSRI and non-SSRI, was associated with a reduced risk of severe complications of COVID-19. Several antipsychotics showed an increased risk for both Sars-CoV-2 infection and severe complications during COVID-19.

**Conclusions:**

The current evidence supports a potential anti-SARS-CoV-2 role for several antidepressants, while the evidence on mood stabilizers or antipsychotics remains controversial. Drug repurposing proved highly successful in response to the current pandemic and psychotropic medications are widely used in clinical practice with well-known safety and tolerability profiles, showing antiviral, immunomodulatory, and anti-inflammatory properties, being perfect candidates for possible treatment of COVID-19. Further research will deliver optimized and specific therapeutic tools that will increase the preparedness of health systems for possible future epidemics.

**Disclosure:**

No significant relationships.

